# COVID-19 Infection Rates in Vaccinated and Unvaccinated Inmates: A Retrospective Cohort Study

**DOI:** 10.7759/cureus.44684

**Published:** 2023-09-04

**Authors:** Luke Ko, Gary Malet, Lisa L Chang, Huu Nguyen, Robert Mayes

**Affiliations:** 1 Biomedical Sciences Pathway Program, California High School, San Ramon, USA; 2 Internal Medicine, California Correctional Healthcare Services, Stockton, USA; 3 College of Education, Governors State University, University Park, USA

**Keywords:** natural immunity, pandemic, breakthrough infections, viral infection, omicron variant, covid-19 vaccination, public health, herd immunity, sars-cov-2 (severe acute respiratory syndrome coronavirus -2), covid-19

## Abstract

Background

In 2023, breakthrough COVID-19 infections among vaccinated individuals and reinfections in previously infected people have become common. Additionally, infections are due to Omicron subvariants of the virus that behave differently from those at the onset of the pandemic. Understanding how vaccination and natural immunity influence COVID-19 infection rates is crucial, especially in high-density congregate settings such as prisons, to inform public health strategies.

Methods

We analyzed COVID-19 surveillance data from January to July 2023 across 33 California state prisons, primarily a male population of 96,201 individuals. We computed the incidence rate of new COVID-19 infections among COVID-bivalent-vaccinated and entirely unvaccinated groups (those not having received either the bivalent or monovalent vaccine).

Results

Our results indicate that the infection rates in the bivalent-vaccinated and entirely unvaccinated groups are 3.24% (95% confidence interval (CI): 3.06-3.42%) and 2.72% (CI: 2.50-2.94%), respectively, with an absolute risk difference of only 0.52%. When the data were filtered for those aged 50 and above, the infection rates were 4.07% (CI: 3.77-4.37%) and 3.1% (CI: 2.46-3.74%), respectively, revealing a mere 0.97% absolute risk difference. Among those aged 65 and above, the infection rates were 6.45% (CI: 5.74-7.16%) and 4.5% (CI: 2.57-6.43%), respectively, with an absolute risk difference of 1.95%.

Conclusion

We note low infection rates in both the vaccinated and unvaccinated groups, with a small absolute difference between the two across age groups. A combination of monovalent and bivalent vaccines and natural infections likely contributed to immunity and a lower level of infection rates compared to the height of the pandemic. It is possible that a degree of 'herd immunity' has been achieved. Yet, using p<0.05 as the threshold for statistical significance, the bivalent-vaccinated group had a slightly but statistically significantly higher infection rate than the unvaccinated group in the statewide category and the age ≥50 years category. However, in the older age category (≥65 years), there was no significant difference in infection rates between the two groups. This suggests that while the bivalent vaccine might offer protection against severe outcomes, it may not significantly reduce the risk of infections entirely. Further research is needed to understand the reasons behind these findings and to consider other factors, such as underlying health conditions. This study underscores the importance of developing vaccines that target residual COVID-19 infections, especially in regard to evolving COVID-19 variants.

## Introduction

Infection rates of COVID-19 have shifted throughout the pandemic due to widespread vaccination, natural infections, and the emergence of novel variants. Although the early phase of the pandemic was characterized by infections in fully susceptible individuals, COVID-19 breakthrough infections among vaccinated individuals and reinfections among previously infected individuals have become increasingly frequent [1-3]. The objective of this study was to explore the impact of vaccination and natural immunity on the current infection rate of COVID-19 in a prison setting. Since the emergence of the highly infectious Omicron variant in December 2021, the United States has observed the development and presence of Omicron subvariants. Determining the impact of vaccination and prior infections on the current rate of Omicron variant infections remains essential for understanding the current infection dynamics of these variants, especially in high-risk transmission settings like prisons. The COVID-19 pandemic has disproportionately affected incarcerated individuals, as the transmission of COVID-19 was high in prison settings, partly fueled by overcrowding, poor or absent ventilation, and introduction from community sources despite high vaccination rates among residents [4].

As of April 18, 2023, the Centers for Disease Control and Prevention (CDC) has simplified COVID-19 vaccine recommendations. It now states that for immunocompetent individuals aged six and over, a single bivalent mRNA vaccine is recommended, regardless of whether they are unvaccinated or have previously received one or more monovalent vaccine doses. Thus, for this age group, the CDC states, "You are up to date when you receive one dose of the updated (bivalent) Pfizer-BioNTech or Moderna COVID-19 vaccine."

Bivalent COVID-19 vaccines became available in September 2022 for adults and adolescents over 12. These "bivalent" vaccines, also called "updated" COVID-19 vaccines, are to be distinguished from the "monovalent" or "original" COVID-19 vaccines, which were previously used as a "primary series" of COVID-19 vaccines. The previous monovalent COVID-19 vaccines are called "original" because they were designed to protect against the original virus that causes COVID-19. As of April 18, 2023, the original, monovalent Pfizer-BioNTech and Moderna COVID-19 vaccines were no longer authorized for use by the U.S. Food and Drug Administration (FDA) in the United States. As of this writing, the updated, bivalent Pfizer-BioNTech and Moderna COVID-19 vaccines are used for all age groups. Current vaccines authorized by the FDA include the following: (1) the Pfizer-BioNTech and Moderna COVID-19 vaccines (discussed above) which are mRNA vaccines; (2) Novavax COVID-19 vaccine, which is a protein subunit vaccine; and (3) J&J/Janssen COVID-19 vaccine, which is a viral vector vaccine, and is no longer available for use in the United States as of May 6, 2023. This paper will discuss the Pfizer-BioNTech and Moderna COVID-19 mRNA vaccines, the vast majority of administered COVID-19 vaccines in the United States, and our studied prison population. The latter two vaccines are either no longer available (J&J/Janssen) or are a minuscule minority of COVID-19 vaccinations administered (Novavax) [5].

Given the recent CDC simplification of COVID-19 vaccination recommendations, our study focuses on the effects of bivalent vaccines on COVID-19 infections from January to July 2023. Of the 96,201 incarcerated individuals studied, 38.1% had received the bivalent vaccine, primarily during the prison system's booster campaign in October-November 2022. A total of 76% of the prison population had received at least two doses of the monovalent primary series vaccine. According to our subgroup analysis, of the 24% who were entirely unvaccinated, 35% of this group had been infected with COVID-19 at some point in the past. Our data indicate that 49.7% of the prison population had experienced a COVID-19 infection at some time. Of the 24% unvaccinated with monovalent vaccines, only 1% chose to receive the bivalent vaccine-a minuscule and insignificant number. Therefore, about 84% of the prison population had developed some level of immunity through vaccination, prior infection, or both. These three groups overlap: monovalent vaccinated, bivalent vaccinated, and those with a history of infection. In this study, we report on COVID-19 infection rates occurring in bivalent-vaccinated individuals (as they are considered 'up-to-date' by CDC guidance) and unvaccinated (to either monovalent or bivalent vaccines) individuals who were incarcerated in a U.S. state prison system during the first six months of 2023. Our findings have broad implications, particularly relevant to incarcerated populations.

## Materials and methods

**Figure 1:**
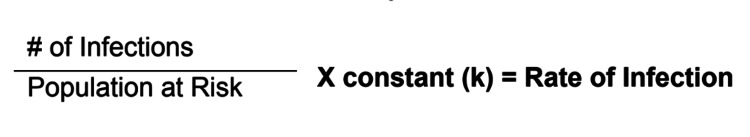
Rate of Infection Calculation

We used a database of anonymized person-level data on COVID-19 testing and COVID-19 vaccination for 96,201 incarcerated individuals in the California state prison system from January 10, 2023, to July 9, 2023. The raw data were available in a pre-created registry, accurately sourced from the prison's electronic health records system, and in a spreadsheet format, which allowed us to sort data using filtering functions. The study's objective was to explore the state of vaccination and natural immunity on the current infection rate of COVID-19, particularly in high-risk populations with intense transmission, such as in prisons. The Omicron variant, which emerged in November 2021, has many lineages and was predominant during our study period. We defined a COVID-positive case as a resident with any conclusive positive SARS-CoV-2 diagnostic test. Most tests were PCR. We classified index cases based on two statuses: COVID-19 bivalent vaccination received and unvaccinated for either the monovalent primary series or bivalent vaccine. Refusals were the reason for non-vaccination the vast majority of the time. We calculated the infection rate, or incidence rate, using the standard formula in Figure 1.

The "number (#) of infections" refers to the index cases identified by surveillance during a defined time frame in a specific population. The "population at risk" includes all individuals in the group or subgroup studied during surveillance. We used 100 as the "k" factor to express the infection rate as a percentage. We performed these calculations first for the prison system as a whole ("statewide"), then filtered the statewide data for those aged 50 and over, and finally for those statewide aged 65 and over. Historically, individuals aged 65 and over have had a higher correlation with severe COVID-19 infections, which influenced our decision to filter the data this way. Among the bivalent-vaccinated, we calculated the infection rate as the number of infections in this group divided by the number of individuals who were bivalent-vaccinated. Similarly, for the unvaccinated, the infection rate was calculated as the number of infections in the unvaccinated group divided by the number of individuals unvaccinated. The sorted data and calculated infection rates are presented in Table 1.

**Table 1:** Infection Rates (Number of COVID-19 Infections/Number of the Population) During January–July 2023 (95% Confidence Interval)

	Statewide		Age ≥ 50		Age ≥ 65	
Bivalent vaccinated	1187/36609	3.24% (CI: 3.06-3.42%)	659/16179	4.07% (CI: 3.77-4.37%)	296/4589	6.45% (CI: 5.74-7.16%)
Unvaccinated	568/20889	2.72% (CI: 2.50-2.94%)	86/2792	3.1% (CI: 2.46-3.74%)	19/442	4.5% (CI: 2.57-6.43%)
Range		0.52%		0.97%		1.95%

All statistical analyses were performed using https://www.wolframalpha.com/. A value of p<0.05 was considered statistically significant. 

A preliminary discussion of the study's limitations is addressed here. (1) Selection bias: the study includes only incarcerated individuals in the California state prison system, which may not represent the general population. This could limit the generalizability of the study's findings. (2) Confounding variables: the study does not control for potential confounding variables such as sex, underlying health conditions, or other factors that could influence the risk of COVID-19 infection. (3) Vaccination status: for those who received the bivalent vaccine, the study does not account for the number of monovalent doses (if any) received prior to the bivalent vaccine, which could affect the vaccine's effectiveness. However, the study did account for the time from bivalent vaccination to the time of the study; approximately 80% of individuals who received the bivalent vaccine did so during the October-November 2022 bivalent vaccination campaign, one to two months before the January start of our study period. The remaining 20% of bivalent-vaccinated individuals received their vaccine during the January-July 2023 period of our study (more details will be provided in the Discussion section).

## Results

**Figure 2:**
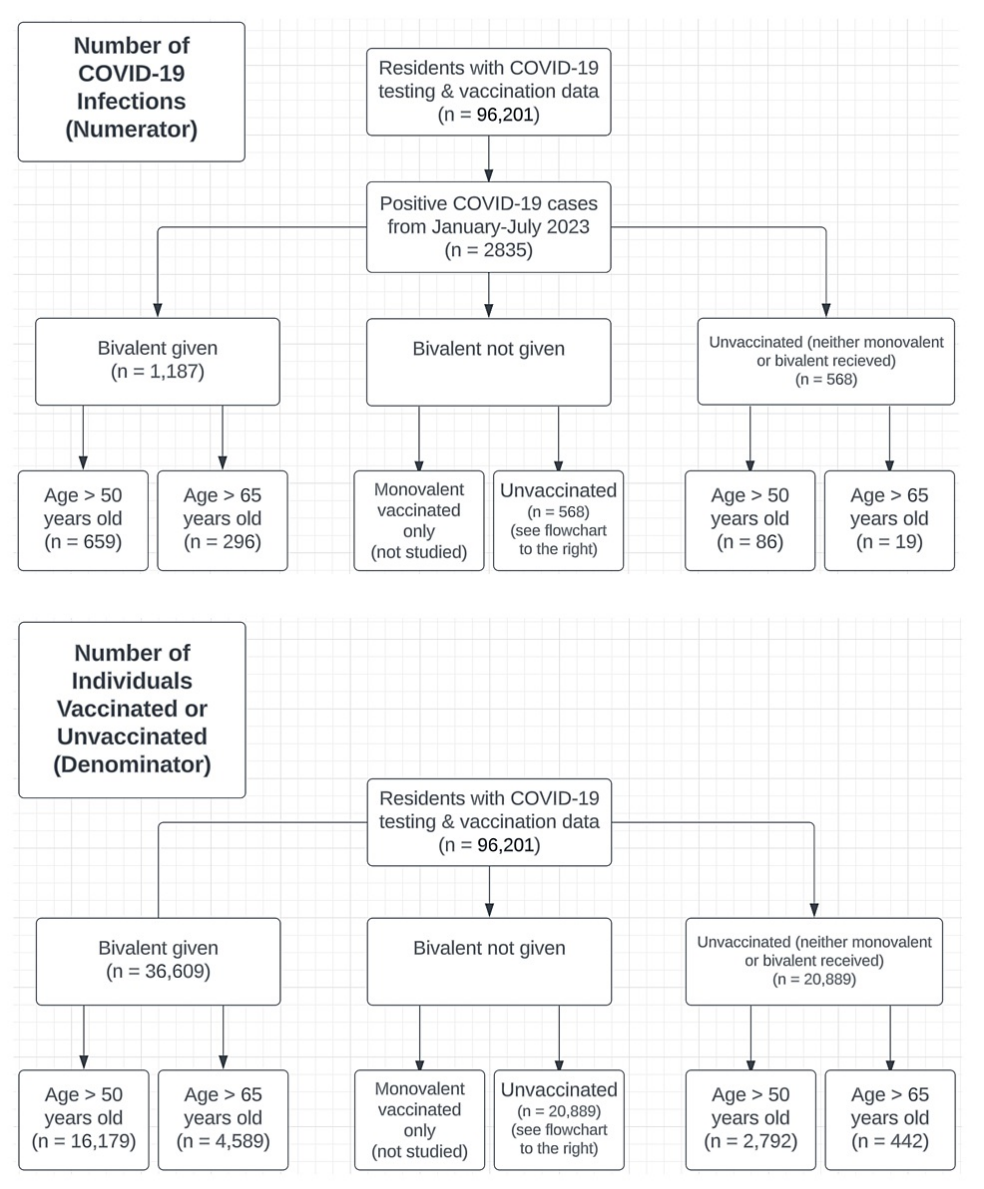
Study Population Flow Chart We obtained data on residents incarcerated in the California state prison system from January 10, 2023, to July 9, 2023, who were diagnosed with COVID-19 based on a positive test. The sample size at each step is plotted in the figure.

We analyzed detailed records of COVID-19 infection and vaccination data from all 33 adult institutions in California’s state prison system (comprising about 96,201 individuals) for the first six months of 2023 (from January 10, 2023, to July 9, 2023) during periods of high-volume testing. The study population was 93% male. We aimed to assess infection rates of the COVID-19 Omicron variant using confirmed index cases, initially grouping cases by their vaccine status, then stratifying them by age 50 and over, then 65 and over, respectively (Figure 2). During this period, COVID-19 mass testing was performed at scheduled intervals for routine surveillance, mass testing in housing units of identified positive cases, and spot testing for self-identified symptoms. There were 2,835 confirmed COVID-19 infections based on the inclusion criteria of having a positive SARS-CoV-2 diagnostic test during these six months. For the prison population as a whole, the incidence rate of new COVID-19 infection amongst COVID-bivalent-vaccinated and entirely unvaccinated was 3.24% (95% confidence interval (CI): 3.06-3.42%) and 2.72% (2.50-2.94%), respectively, with an absolute risk difference of only 0.52%. When data were filtered for those aged 50 and above, the infection rates were 4.07% (3.77-4.37%) and 3.1% (2.46-3.74%), respectively, revealing a mere 0.97% absolute risk difference. Among those aged 65 and above, infection rates were 6.45% (5.74-7.16%) and 4.5% (2.57-6.43%), respectively, with an absolute risk difference of 1.95%. The absolute difference in infection rates is small within each age category.

The calculated p-values for the comparison between the bivalent vaccinated and unvaccinated groups for each age category are the following: Statewide, p = 0.000489; age ≥ 50 years, p = 0.0148; age ≥ 65 years, p = 0.1065.

## Discussion

Using detailed epidemiologic data from COVID-19 surveillance within the California state prison system, we note low infection rates in both the vaccinated and unvaccinated populations, with a slight absolute difference between the two across age groups during an Omicron-variant-predominant period. Using p<0.05 as considered statistically significant, the bivalent vaccinated group showed slightly, but statistically significant, higher infection rates compared to the unvaccinated group in the statewide category and the age ≥50 years category. In the older age group (≥65 years), there was no significant difference in infection rates between the two groups. However, the unvaccinated population in this group was notably smaller and likely not sufficiently powered.

All COVID-19 infections in vaccinated individuals are termed 'breakthrough' infections. A significant 41.9% of the infections during our 6-month study were bivalent vaccine breakthrough infections (1,187 infections were in bivalent-vaccinated individuals out of 2,835 infections during these six months, or 1,187/2,835). An even greater 75% are breakthrough infections if defined as infections in those who completed two shots of the monovalent series.

**Figure 3:**
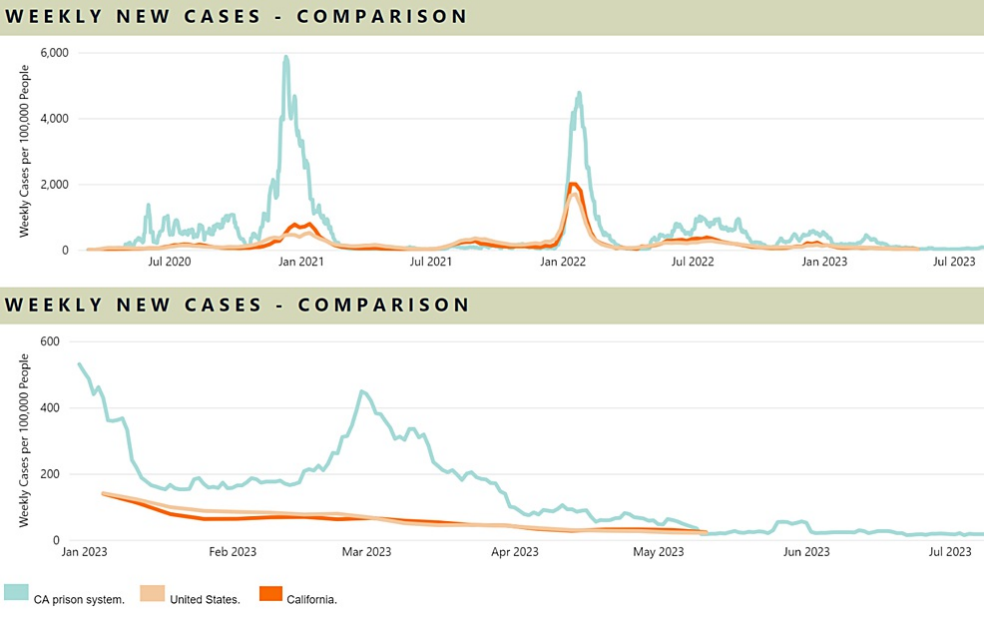
California State Prison COVID-19 Trends Courtesy of https://www.cdcr.ca.gov/covid19/population-status-tracking.

Prior studies showed high efficacy of COVID-19 vaccines in reducing infection and transmission rates [4], which is why our results are perplexing. The vaccinated and unvaccinated had similar absolute infection rates; the vaccinated had slightly but statistically significantly higher infection rates. Our results may be influenced by the current immunologic state of the studied population, not solely by the efficacy of the COVID-19 bivalent (or monovalent) vaccines. As noted earlier, approximately 84% of the prison population had developed some immunity through a patchwork of administered monovalent vaccines, administered bivalent vaccines, naturally acquired COVID-19 infection, or any combination of these. From January 2023 to July 2023, in the fourth year of the COVID-19 pandemic, there were historically low levels of circulating COVID-19 virus in the prison system (Figure 3). This may partially explain the similarities in infection rates between the vaccinated and the unvaccinated, where the differentiating effects of vaccination status become comparatively less significant. This contrasts with the early pandemic, where overall cases were high, occurring in fully susceptible individuals, and infection rates between the vaccinated and unvaccinated differed significantly-being higher in the latter and lower in the former. This study supports the benefits of COVID-19 vaccination at a population level, especially in vulnerable, high-density congregate settings.

 

Several factors may have enhanced our ability to observe statistically meaningful findings in the present study. The risk of transmission among close-proximity contacts in the prison setting and consistency in contact structure, especially in light of the increased transmissibility of the Omicron COVID-19 variant-may have enhanced statistical power in our sample. The strengths of this study include access to records of prior COVID-19 vaccine receipt and dates of COVID-19 infection (based on frequent testing throughout the pandemic) for all residents of the California state prison system. The large sample size facilitates analysis of the contribution (or lack thereof) of prior vaccination to the risk of infection.

The two available bivalent COVID vaccines by Pfizer-BioNTech and Moderna protect against both the original virus that causes COVID-19 and the Omicron variant BA.4 and BA.5. As of April 18, 2023, the CDC has simplified COVID-19 vaccine recommendations, stating that for immunocompetent individuals age six and over, a single bivalent mRNA vaccine is recommended, regardless of whether they are unvaccinated or had previously received one or more monovalent vaccine doses. Thus, for this age group, the CDC states, "You are up to date when you get one updated (bivalent) Pfizer-BioNTech or Moderna COVID-19 vaccine" [6].

The primary antigenic target for COVID-19 vaccines is the large surface spike protein, which binds to the angiotensin-converting enzyme 2 (ACE2) receptor on host cells. Monovalent vaccines encode or contain antigens from the original SARS-CoV-2 strain, whereas bivalent vaccines include antigens from an Omicron variant. Vaccines available in the United States substantially reduce the risk of COVID-19, especially severe/critical disease, and have been associated with substantial reductions in COVID-19-associated hospitalizations and deaths, even in the context of variants that partially evade vaccine-induced immune responses. Although effectiveness wanes, vaccine-induced immunity continues to reduce the risk of severe disease, and repeat vaccination is associated with a relative increase in efficacy over several months.

As of May 10, 2023, CDC data estimate that 69.5% of the U.S. population has completed at least two shots of the original, monovalent COVID-19 primary series. Furthermore, it is estimated that 17.0% of the U.S. population has received the updated bivalent vaccine [5].

The clinical effectiveness of bivalent vaccines is supported by observational data, indicating a moderate level of protection against infection depending on the time elapsed since the last vaccine dose [7-9]. Indirect evidence from monovalent trials and observational studies supports the use of bivalent vaccines and suggests their high efficacy as both primary series and booster doses [10-12]. Using a bivalent vaccine is comparable to updating seasonal influenza vaccines, where new vaccines are produced annually to match the circulating virus strains. Bivalent vaccines are expected to be at least as effective as monovalent vaccines [10-12].

Multiple observational studies have indicated that vaccine protection against COVID-19 infection wanes over time, after primary series and booster vaccinations, and with monovalent and bivalent vaccines in children and adults [13-15]. Protection against hospitalization and death also wanes somewhat but less than protection against infection [16-18].

The risk of breakthrough infections with specific variants, such as Delta and Omicron, is higher compared to earlier variants of COVID-19. However, the risk of severe breakthrough infection with the Omicron variant remains low, especially among individuals who have received a booster vaccine dose. Observational data also indicates that breakthrough infections are associated with a lower number of symptoms, shorter duration of symptoms, a lower likelihood of persistent symptoms lasting more than 28 days (referred to as "long COVID-19"), and a higher chance of asymptomatic infection compared to infections in unvaccinated individuals [19-21]. Previous data collected before the emergence of the Omicron variant suggested that individuals who experienced breakthrough infections despite vaccination may also have a lower likelihood of transmitting the virus to others [22,23].

Although the impact of vaccination on transmission in the context of Omicron infection is still being investigated, vaccination is expected to play a role in reducing the likelihood of transmission even with this variant. Vaccination remains crucial in mitigating the spread of COVID-19 and reducing the severity of breakthrough infections. The following limitations should also be considered.

Generalizability

The study is limited to incarcerated individuals in the California state prison system, which may not represent the general population. The incarcerated population lacks internet access and likely has differing health literacy and views on COVID-19 vaccines compared to the public.

Confounding variables

There is the possibility of confounding between individuals who are vaccinated and those who are not against COVID-19. The choice to vaccinate or not may self-select for differences in an individual's behaviors and attitudes relating to other COVID-19 risk-reduction strategies (for example, whether or not to wear masks and whether or not to practice social distancing). Such self-selection can occur either positively or negatively (i.e., to be more cautious versus more risk-taking after vaccination).

Possible underreporting of COVID-19 symptoms

Anecdotal reports suggest that some inmates choose not to report positive COVID-19 symptoms or refuse surveillance testing to avoid temporary isolation following a positive test. The prevalence of these attitudes is unknown but could skew the study results.

Incomplete observation period

A minority of individuals chose not to be vaccinated during the prison booster campaign from October to November 2022 but were vaccinated during the January-to-July 2023, six-month study period. The study will not capture a six-month observation of the vaccine effect on these individuals. We estimate that about 20% of our bivalent-vaccinated individuals fall into this category.

Inability to calculate reinfection rates

The study could not calculate reinfection rates for those with a previous history of COVID-19 infection, as our database only reflected one date of infection, if any.

Inability to subgroup for breakthrough reinfections

The study was unable to subgroup for "breakthrough reinfections," that is, new infections in previously vaccinated and previously COVID-19-infected individuals.

Lack of data on hospitalizations

We do not have data on how many (if any) COVID-19 infections led to hospitalizations during our six-month study, but we expect the number to be minuscule. According to available data, 1,110 of 96,201 individuals had COVID-related hospitalizations at any time in the past (including the six months of our study), which is 1.2% of our study population. With diminishing infection rates during our study's six months relative to past years (Figure 3) and the higher proportion of vaccinated individuals, the incidence of COVID-19 hospitalizations during our study period should, as a result, be a tiny fraction of 1.2%.

Vaccination has reduced infection rates to historically low levels in our large prison population. Of these, 76% completed two doses of the monovalent vaccine, and 38.1% received the up-to-date bivalent vaccine. Additionally, 49.7% had a COVID-19 infection at some point in the past. Thus, 84% of the prison population has developed some level of immunity through either vaccination or infection. Anthony Fauci, M.D., former Chief Medical Advisor to the President of the United States, had predicted that between 70% and 85% of the U.S. population might need to be vaccinated to achieve 'herd immunity' against COVID-19. There have been debates about whether herd immunity can ever be achieved for COVID-19, particularly when compared to diseases like polio and measles, for which herd immunity has been successfully established in the United States [24]. In a population with sufficient immunity, herd immunity indirectly protects those who are not immune by minimizing the likelihood of effective contact between a susceptible individual and an infected host. Even once achieved, the efficacy of herd immunity depends on the duration and potency of the acquired immunity. In cases where lifelong immunity is induced, such as with measles, through either vaccination or infection, herd immunity becomes highly effective at halting pathogen transmission within the population. If the vaccination does not confer robust immunity to all recipients, the immunization threshold required to protect the population increases [25]. Like with pertussis, immunity against COVID-19 wanes over time or is incomplete, making herd immunity less effective and allowing for periodic breakthrough infections-and outbreaks-to occur [26].

## Conclusions

The COVID-bivalent vaccinated group showed a higher infection rate than the unvaccinated group in the statewide category and the age ≥50 years category. However, the absolute difference in infection rates is negligible. In the older age group (≥65 years), there was no significant difference in infection rates between the two groups. However, the unvaccinated population in this group was notably smaller and likely not sufficiently powered. This study suggests that while the bivalent vaccine might offer protection against severe outcomes, it may not significantly reduce the risk of overall infections. Further research is needed to understand the reasons behind these findings and to consider other factors, such as underlying health conditions. This study underscores the importance of developing vaccines targeting residual COVID-19 infections, especially regarding evolving COVID-19 variants.
